# Regulation of Tissue Factor by CD44 Supports Coagulant Activity in Breast Tumor Cells

**DOI:** 10.3390/cancers14133288

**Published:** 2022-07-05

**Authors:** Amélie V. Villard, Anthony Genna, Justine Lambert, Marianna Volpert, Agnès Noël, Brett Hollier, Myriam Polette, Aline M. Vanwynsberghe, Christine Gilles

**Affiliations:** 1 Laboratory of Tumor and Development Biology, GIGA-Cancer, University of Liège, 4000 Liège, Belgium; amelie.villard@uliege.be (A.V.V.); anthony.genna@uliege.be (A.G.); justine.lambert26@gmail.com (J.L.); agnes.noel@uliege.be (A.N.); vanwynsberghe.aline@gmail.com (A.M.V.); 2 Australian Prostate Cancer Research Centre-Queensland (APCRC-Q), School of Biomedical Sciences, Faculty of Health, Queensland University of Technology, Princess Alexandra Hospital, Translational Research Institute, Brisbane, QLD 4102, Australia; marianna.volpert@qut.edu.au (M.V.); b.hollier@qut.edu.au (B.H.); 3 Université de Reims Champagne-Ardenne, Inserm, P3Cell UMR-S1250, SFR CAP-SANTE, and Laboratoire de Biopathologie, CHU Reims, Hôpital Maison Blanche, 51092 Reims, France; myriam.polette@univ-reims.fr

**Keywords:** tissue factor, CD44, epithelial-mesenchymal transitions, metastasis, coagulation

## Abstract

**Simple Summary:**

Metastasis and thromboembolic complications are the main cause of cancer-associated death. An overexpression of coagulation factors, and particularly Tissue factor, by tumor cells is a key event implicated in this observed hypercoagulability. Tissue Factor is indeed a cellular initiator of the coagulation cascade which has been associated with aggressive tumor phenotypes such as those characteristic of Epithelial-Mesenchymal Transitions (EMTs) and Cancer Stem Cells (CSCs). Understanding molecular mechanisms controlling Tissue Factor overexpression in those tumor phenotypes is thus an important aspect of cancer research. We show here that CD44 (a transmembrane marker of CSC and EMT phenotypes) contributes to regulate TF expression at a transcriptional level, thereby supporting procoagulant properties in tumor cells that facilitate their metastatic spread.

**Abstract:**

Previous work identified Tissue Factor (TF), a key activator of the coagulation cascade, as a gene induced in cellular contexts of Epithelial-Mesenchymal Transitions (EMTs), providing EMT^+^ Circulating Tumor Cells (CTCs) with coagulant properties that facilitate their metastatic seeding. Deciphering further molecular aspects of TF regulation in tumor cells, we report here that CD44 and TF coexpress in EMT contexts, and that CD44 acts as a regulator of TF expression supporting procoagulant properties and metastatic seeding. A transcriptional regulatory mechanism bridging CD44 to TF expression was further evidenced. Comparing different TF –promoter luciferase reporter constructs, we indeed found that the shortest -111 pb TF promoter fragment harboring three Specificity Protein 1 (Sp1) binding sites is still responsive to CD44 silencing. The observation that (i) mutation within Sp1 binding sites decreased the basal activity of the -111 pb TF promoter construct, (ii) CD44 silencing decreased Sp1 protein and mRNA levels and (iii) Sp1 silencing diminished TF expression further points to Sp1 as a key mediator linking CD44 to TF regulation. All together, these data thus report a transcriptional regulatory mechanism of TF expression by CD44 supporting procoagulant activity and metastatic competence of CTCs.

## 1. Introduction

Circulating tumor cells (CTCs) represent a heterogeneous population containing metastatic founders [1,2,3,4,5]. Epithelial-Mesenchymal Transitions (EMTs) are recognized key processes in the biology of CTCs, in line with massive experimental data demonstrating that EMTs provide tumor cells with enhanced invasive, survival, stemness and niching properties [6,7,8,9,10]. Accordingly, EMT molecular actors have been extensively reported in CTCs isolated from most types of cancers [5,11,12,13]. Nevertheless, molecular mechanisms enabling few CTCs to survive in the bloodstream and to initiate metastatic niches at secondary sites remain misunderstood.

Hypercoagulability is a long-known correlate of malignancy (Trousseau’s syndrome) [14], and data implicating coagulation-dependent mechanisms in metastasis are accumulating. Tissue Factor (TF), a transmembrane protein overexpressed by a variety of tumor cells, has emerged as a central player linking coagulation and cancer [14,15,16,17,18]. Schematically, TF serves as a receptor for factor VIIa (FVIIa), and thereby triggers the successive activation of downstream coagulation factors and the generation of fibrin. Although TF also initiates signalling events that facilitate tumor progression in coagulation-independent ways [18,19], a determinant role of TF-associated coagulation mechanisms in supporting metastasis has been demonstrated. A local activation of coagulation is indeed considered to contribute to the generation of a pericellular fibrin/platelet-rich cocoon protecting CTCs against shear stress, anoikis and immune attack, and also providing a favorable niche for early metastatic seeding [14,15,16,17,20,21,22].

In this context, understanding molecular mechanisms driving TF expression in tumor cells seems crucial. Previously, we reported EMT-driven regulatory mechanisms leading to the overexpression of TF and providing EMT+ tumor cells with procoagulant properties facilitating early steps of the metastatic colonization in experimental metastasis assays [23,24]. Pursuing this line of research, we examine here the possibility that CD44 could directly contribute to TF regulation and thereby modulate coagulant activity on EMT+ tumor cells. CD44 is a multifunctional transmembrane glycoprotein that has been implicated in a variety of pro-tumoral and pro-metastatic functions [25,26,27,28]. CD44 has thus been narrowly associated to EMT, either as an EMT inducer but also, inversely, as an EMT target gene. Since the pioneer study of Pr. Clarke’s group [29], CD44 has also been demonstrated to be a marker of Cancer Stem Cell (CSC) phenotypes in solid tumors [30]. More than just being a marker, CD44 also plays functional roles in CSC properties, many of which are also recognized EMT-driven properties, including self-renewal, plasticity, tumor initiation, metastasis, and chemo/radio resistance [28,30,31]. More particularly, as a membrane receptor, CD44 is structurally involved in cell-cell or cell-substrate interactions that facilitate various steps of the metastatic colonization. If hyaluronic acid is its most well-known ligand, CD44 also binds several matrix proteins and acts as a co-receptor for many receptor tyrosine kinases (RTKs) (including c-Met and the Epidermal Growth Factor Receptor (EGFR)). CD44 is accordingly a central signaling molecule, modulating various downstream signaling pathways (including ERK, PI3K, SRC, AKT, RAS-MAPK) [32,33]. In some cellular contexts, a γ-secretase-dependent liberation of a CD44 bioactive intracellular domain (ICD), occurring sequentially after the shedding of the extracellular domain, has also been reported [34,35]. Adding complexity to the understanding of CD44 involvement in metastasis, CD44 is subjected to complex and numerous alternative splicing patterns that diversify its structure and functions, but that are still largely misunderstood. The first five and the last four exons of the human CD44 gene are constant and encode the shortest CD44 standard isoform (CD44s). The central exons can be alternatively spliced, generating numerous variant isoforms (referred to as CD44v) harboring a variable region within the extracellular domain [25,26,27,28].

Considering our previous work supporting an EMT/TF/coagulation axis favoring metastasis and the narrow relationships between CD44 and EMT, we examined here the impact of CD44 in this axis. We thus report that CD44 may impact on TF expression at a transcriptional level and modulate the coagulant activity of EMT^+^ breast tumor cells.

## 2. Material and Methods

Supplementary Table S1 shows an overview of the study design.

### 2.1. Cell Culture

Human breast cancer cell lines (MCF7, T47D, MDA-MB-468, MDA-MB-231 and Hs578T) were obtained from the ATCC (American Type Culture Collection). MDA-MB-468 iSnail cells were generated by transducing the doxycycline-inducible pINDUCER20 lentiviral expression construct harboring Snail cDNA (or GFP as a control) [36,37]. All cell lines were used within 10 passages after authentication (STR DNA typing, Leibniz-Institute DSMZ), and were mycoplasma free. Cells were cultured with DMEM medium (Gibco, Thermo Fisher Scientific, Waltham, MA, USA) supplemented with 10% FBS (Sigma Life Science, Saint-Louis, MO, USA) and 1% Penicillin-Streptomycin (Gibco, Thermo Fisher Scientific, Waltham, MA, USA). To induce EMT, MDA-MB-468 were cultured with 20 ng/mL of recombinant EGF (Sigma-Aldrich, Saint-Louis, MO, USA) or 125 ng/mL of doxycycline (Sigma Life Science, for the iSnail model). For mithramycin treatments, MDA-MB-231 and Hs578T were incubated for 48 h and 24 h respectively with 100 nM of mithramycin (Thermo Fisher Scientific).

### 2.2. siRNA Transfection

Cells were transfected with RNAiMax (Invitrogen, Thermo Fisher Scientific) and 20 nM of the siRNA duplexes 24 h after plating. Specific 19-nt sequences were used to generate 21-nt sense and 21-nt antisense strands of the type (19N) TT (N, any nucleotide). Pools of three siRNA duplexes were used (for targeting siRNAs and control siRNA). siRNA sequences were purchased from Eurogentec (Liège, Belgium) and are listed in Supplementary Table S2. Cells were harvested for subsequent analyses 48 h after transfection.

### 2.3. RT-qPCR, Western Blotting Analyses and Flow Cytometry Detection of Cell-Surface TF

RT-qPCR was performed on RNA extracted from cell cultures with a High Pure RNA Isolation Kit, reverse transcribed using the First Strand cDNA Synthesis kit and amplified on the LightCycler480 with the Universal Probe Library system (all kits are from Roche, Basel, Switzerland). Primer sequences are provided in Supplementary Table S3. Expression levels of genes of interest were quantified with the 2^−∆∆C^ method and results are expressed as fold change compared to the control condition.

For western blotting analyses, total proteins were separated on 10% SDS-polyacrylamide electrophoresis gels and transferred to PVDF membranes. The antibodies used are listed in Supplementary Table S4. The original uncropped images of the blots and densitometric analyses are provided as Supplementary Materials.

For the detection of cell surface-associated TF and CD44, cells were detached with trypsin-EDTA, labelled with the appropriate antibody (Supplementary Table S5) and analysed with the BD LSRFortessa™ Flow Cytometer (BD Biosciences, Erembodegem, Belgium). Results were analyzed with FlowJo software (TreeStar, OR, USA).

### 2.4. Sequencing of CD44 Isoforms

Endpoint RT-PCR was performed on RNA extracted from cell cultures with the High Pure RNA Isolation Kit, reverse transcribed using the First Strand cDNA Synthesis kit and amplified using a forward primer located in the fifth exon and a reverse primer located in the nineteenth exon of the human CD44 gene for 40 cycles (all kits are from Roche, Basel, Switzerland). The sequences of the primers are provided in Supplementary Table S6. PCR products were analysed on agarose gel. Major PCR products were extracted from the gel using Wizard^®^ SV Gel and PCR Clean-Up System (Promega) and sequenced at the GIGA-genomics platform (ULiege) using the same primers.

### 2.5. Enzymatic Coagulation Assays

The fluorescent enzymatic coagulation assay was adapted from Rothmeier and colleagues [38]. Briefly, cells were seeded in 96 well-opaque plates. Twenty-four hours after, cells were washed with HBS (10 mM HEPES [pH 7.4], 137 mM NaCl, 5.3 mM KCl, 1.5 mM CaCl_2_) and incubated with 100 μL of HBS containing FVIIa (0.5 nM—Haematologic Technologies, Essex Junction, VT, USA), Factor X (FX) (50 nM—Haematologic Technologies, Essex Junction, VT, USA) and a fluorogenic peptide substrate I-1100 (100 µM—Bachem, Budendorf, Switzerland) of activated FX (FXa). The kinetic of fluorescence release after peptide cleavage was measured during 8 h with SpectraMax^®^ i3x (Molecular Device, CA, USA) at an emission wavelength of 340 nm and an excitation wavelength of 440 nm.

### 2.6. Visual Clotting Assays

Clotting assays were described previously [23]. Briefly, cells were suspended in 600 µL of serum-free DMEM (providing a calcium concentration of 1.2 mM compatible with coagulation) and mixed with 300 µL of whole blood collected from healthy donors on 3.2% sodium citrate. Clot formation time was monitored. All clotting experiments were performed at least three times during an observation period of 4 h. Due to interpersonal variability regarding clotting time, results from one representative experiment are shown.

### 2.7. Dual-Luciferase Reporter Assays

Cells were seeded, in triplicate, into 24 well plates for 24 h and transfected with lipofectamine 2000 reagent (Invitrogen, Thermo Fisher Scientific) and 1.7 µg/well of the TF promoter reporter constructs and 13.6 ng/well of the Renilla luciferase reporter phRG-TK (Promega, Madison, WI, USA) were used as an internal control. The firefly luciferase:renilla luciferase activity ratio was measured 24 h after transfection using the Dual-Luciferase Reporter Assay System^®^ (Promega, Madison, WI, USA). The series of TF promoter reporter constructs were generated in Pr. Mackman’s laboratory [39] and obtained from Addgene (Watertown, MA, USA) (Supplementary Table S7). A -111 pb TF promoter construct mutated within all three overlapping Sp1/EGR-1 binding sites was generated in pGL3 (Promega) according to a mutation described in [40], GGGCGG: TTTATT, through GenScript, New Jersey.

### 2.8. Mice Models

All animal studies were approved by the Animal Ethics Committee of the University of Liège (n° 1932, ULiège, Belgium). BALB/c (7 weeks of age) were purchased from Charles River Laboratories (Wilmington, MA). After siRNA transfection, cells (1 × 10^5^ cells per mouse) were injected in the tail vein. To quantify early metastatic seeding, mice were sacrificed 24 h after injection and lungs were collected. After tissue disruption in MagNa lyser Green beads tubes (Roche), total RNA was extracted from lungs with a NucleoSpin RNA Midi kit (Macherey-Nagel, Düren, Germany). Human GAPDH levels were quantified by RT-nested qPCR, as previously described [23,41], to evaluate tumor cell contents in the lungs. In parallel, murine GAPDH was amplified from the same reverse-transcribed RNA using specific murine GAPDH primers. The sequences of the primers used are provided in Supplementary Table S3. Human GAPDH levels were normalized to the corresponding murine GAPDH levels for each mouse. Values are normalized to mean expression in reference group in order to combine independent mice experiments.

### 2.9. Statistical Analysis

Results are expressed as mean +/− SEM. Statistical analyses were performed with GraphPad Prism software version 8.3.0 (GraphPad software, San Diego, CA, USA). In vitro RT-qPCR results, promoter reporter assay results, and MFI results were expressed as fold change and were analyzed with a two-tailed one sample *t* test. In vivo results were analyzed with a one-tailed Mann–Whitney test. A *p* < 0.05 was considered statistically significant. * *p* < 0.05, ** *p* < 0.01, *** *p* < 0.001, **** *p* < 0.0001.

## 3. Results

### 3.1. CD44 and TF Are Concomitantly Expressed in EMT^+^ Cells and CD44 Silencing Decreases TF Expression

We have previously identified Tissue Factor as a target gene of EMTs providing EMT^+^ cells with enhanced coagulant properties and favoring metastatic in experimental metastasis assays [23,24]. Deciphering further EMT-associated mechanisms controlling TF expression in tumor cells, we examined here a potential impact of CD44 in TF regulation.

**Figure 1:**
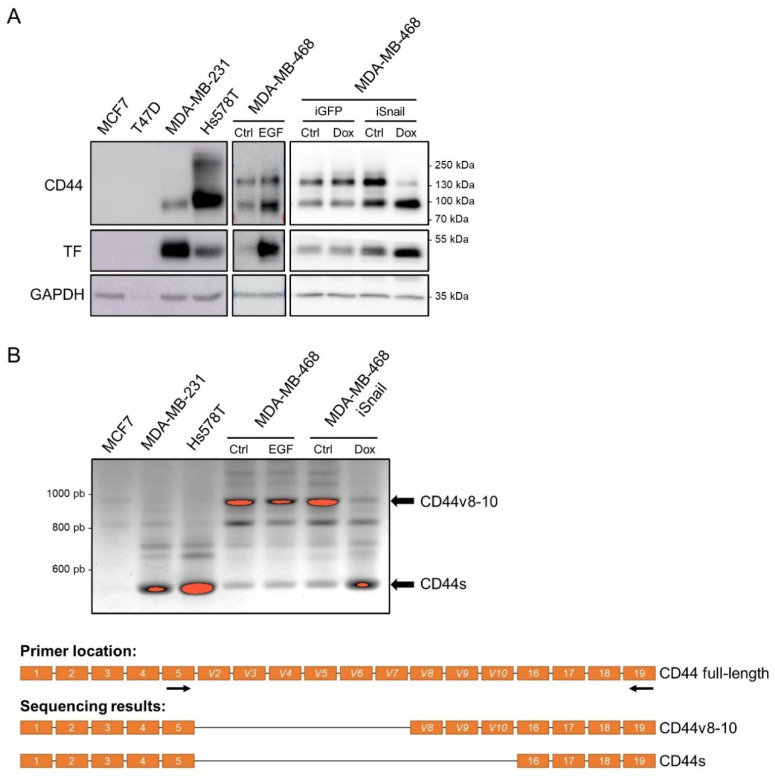
TF and CD44 coexpress in EMT cellular contexts: (**A**) Western blotting analyses of CD44, Tissue Factor (TF) and GAPDH in Epithelial-Mesenchymal Transition-positive (EMT^+^: MDA-MB-231 and Hs578T), EMT-negative (EMT^−^: MCF7 and T47D) and EMT-inducible models: MDA-MB-468 treated or not (Ctrl) with EGF and MDA-MB-468 iSnail or its control iGFP treated or not (Ctrl) with doxycycline (original western blot images are shown in Supplementary Materials). (**B**) Sequencing analyses of major CD44 isoforms present in different cell lines as indicated. An end-point RT-PCR (top panel) using a forward primer located in the fifth exon and a reverse primer located in the nineteenth exon of the human CD44 gene (as shown in the schematic representation in the bottom panel) was performed. The two major amplicons (appearing as saturated bands in red on the gel) were extracted from the gel and sequenced. The sequencing results clearly revealed two major isoforms (CD44v8-10 and CD44s) as indicated on the cartoon. (Figure S3: Uncropped Western blot figures).

To address this question, we first examined CD44 and TF expression in different EMT cellular models that previously allowed us to associate TF expression to EMTs [23,24]: (i) in well-known invasive EMT^+^ MDA-MB-231 and Hs578T versus non-invasive EMT^−^ MCF7 and T47D human breast tumor cell lines, (ii) in MDA-MB-468 known to be induced to EMT by EGF and (iii) in the MDA-MB-468 cells expressing a doxycycline-inducible vector for Snail (MDA-MB-468-iSnail) in which we previously reported an induction of TF associated to EMT after doxycycline treatment [23]. Western blotting analyses clearly revealed a concomitant expression of TF and of a ~90 kDa form of CD44 in EMT^+^ cellular contexts (Figure 1A). These analyses also revealed different CD44 western blot patterns between the different EMT^+^ cell lines. In MDA-MB-231 cells, CD44 was mostly found expressed as this ~90 kDa form. Hs578T showed a strong expression of a similar molecular weight form of CD44 although higher molecular weight bands were clearly detected. In the MDA-MB-468, a high molecular weight form of ~130 kDa and a similar ~90 kDa molecular weight form were observed. After EGF induction, both forms increased. Very interestingly, after iSnail-doxycycline induction, an apparent switch from the higher molecular weight form of ~130 kDa to the ~90 kDa molecular weight form was observed (Figure 1A). Although it is outside the scope of this study to perform a detailed analysis of CD44 variants in our different models, we performed end-point PCR using a pair of primers encompassing the variable region (Figure 1B) which showed a major product in all EMT^+^ models of ~590 pb. Sequencing analysis of the PCR product purified after excision from the agarose gel revealed that this band corresponded to CD44s. Interestingly, a shift towards this variant was particularly obvious in the MDA-MB-468 iSnail model. These data obtained by sequencing also support the idea that the ~90 kDa molecular weight form observed in western blotting corresponds to CD44s and thus suggest that TF particularly associates with CD44s. A concomitant expression of CD44 and TF in EMT^+^ contexts was also confirmed by FACS analyses (Supplementary Figure S1).

**Figure 2:**
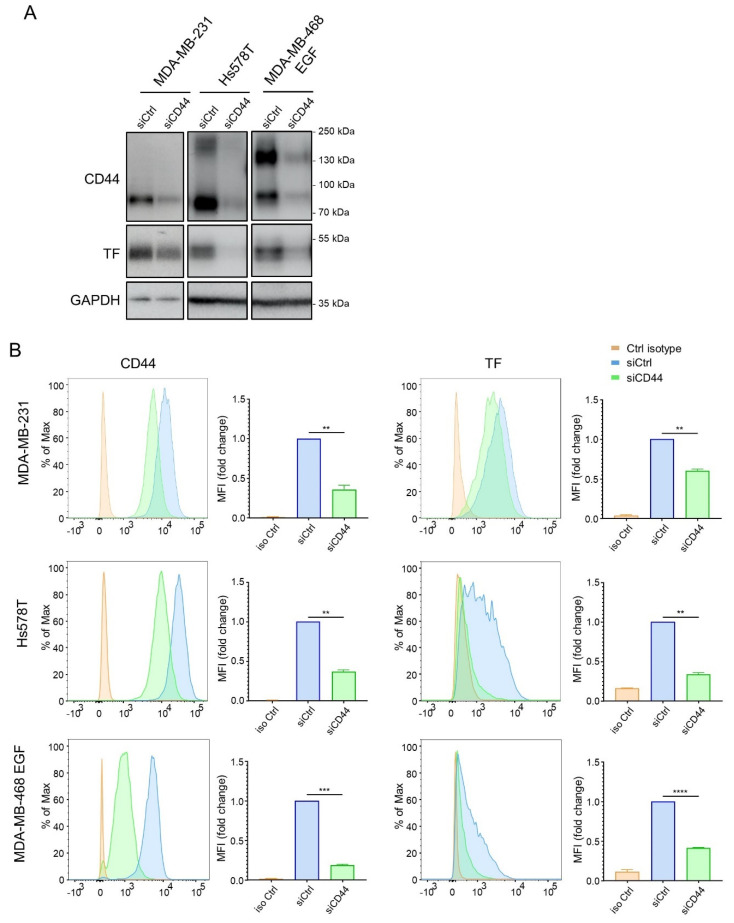
CD44 silencing decreases TF expression: (**A**) Western blotting analyses of CD44, TF and GAPDH in EMT^+^ MDA-MB-231, Hs578T and EGF-treated MDA-MB-468, and transfected with a pool of 3 siRNAs directed against CD44 (siCD44) or 3 non-targeting siRNAs (siCtrl) (original western blot images are shown in Supplementary Materials) (**B**) FACS analyses and associated Mean Fluorescence Intensity (MFI) quantifications of CD44 and TF in cellular models described in (**A**). **, *p* < 0.01; ***, *p* < 0.001; ****, *p* < 0.0001. (Figure S3: Uncropped Western blot figures).

To further explore a potential regulatory relationship between CD44 and TF, we next examined the impact of CD44 silencing on TF expression. In EMT^+^ cellular models (MDA-MB-231, Hs578T and EGF-treated MDA-MB-468), CD44 silencing clearly diminished TF protein levels (Figure 2A). Because cell surface TF is determinant for its coagulant activity, we also verified by FACS analyses that CD44 silencing decreased cell surface levels of TF (Figure 2B). Although FACS results for TF showed a heterogeneous distribution among cells, the Mean Fluorescence Intensity (MFI) quantification showed a clear decrease in TF levels after CD44 silencing (Figure 2B).

### 3.2. CD44 Silencing Hinders Coagulant Properties and Metastatic Seeding of EMT^+^ Cells

**Figure 3:**
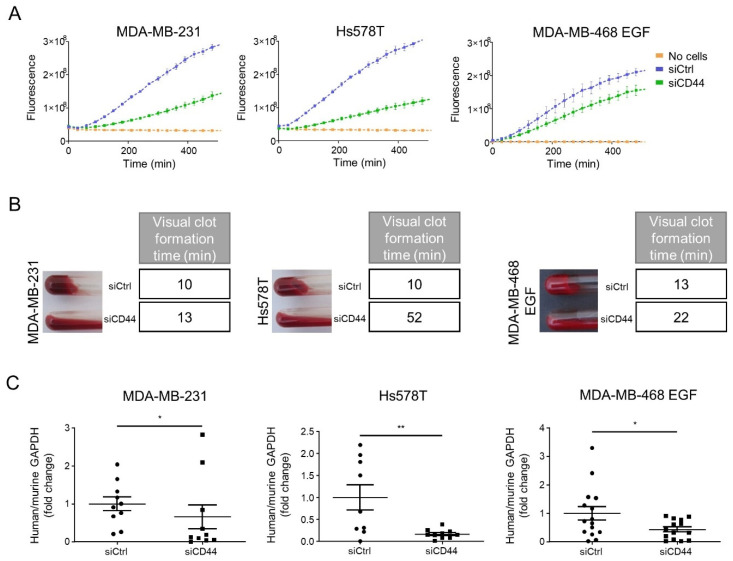
CD44 silencing decreases in vitro coagulant properties and metastatic seeding in vivo: (**A**) Enzymatic fluorescent coagulation assays and (**B**) visual clot assays performed with EMT^+^ MDA-MB-231, Hs578T and EGF-treated MDA-MB-468, and transfected with a pool of 3 siRNAs directed against CD44 (siCD44) or 3 non-targeting siRNAs (siCtrl). (**C**) To quantify metastatic seeding, RT-nested qPCR for human GAPDH was performed on total RNA extracted from the lungs of mice injected intravenously for 24 h with MDA-MB-231, Hs578T or MDA-MB-468 induced to EMT by EGF, and silenced (siCD44) or not (siCtrl) for CD44 in vitro before injection. Results are expressed as the ratio of human GAPDH (normalized to mouse GAPDH) for each mouse to the mean value of the reference group (siCtrl). Two independent experiments were pooled: MDA-MB-231 (siCtrl *n* = 10, siCD44 *n* = 10), Hs578T (siCtrl *n* = 9, siCD44 *n* = 10) and MDA-MB-468 treated with EGF (siCtrl *n* = 15, siCD44 *n* = 14). *, *p* < 0.05; **, *p* < 0.01.

Considering this newly identified regulation of TF by CD44, we further examined the impact of CD44 silencing on coagulant properties. To do so, we optimized an in vitro fluorogenic coagulation substrate assay (adapted from [38]) in which cells are incubated with recombinant FVIIa and FX combined with a fluorescent peptide substrate of FXa. We first validated this assay by a comparison of EMT^+^ and EMT^−^ cells that confirmed a higher coagulant activity of EMT^+^/TF^+^ cell lines (Supplementary Figure S2). We also verified the implication of TF in this enzymatic coagulation assay using MDA-MB-231 cells silenced for TF (MDA-MB-231shTF) previously generated in the laboratory [23]) which clearly displayed a lower activity on the fluorescent peptide substrate (Supplementary Figure S2). Using this assay, we were thus able to show that silencing CD44 in EMT^+^ MDA-MB-231, Hs578T and in EGF-treated MDA-MB-468, resulted in decreased coagulant activity (Figure 3A). This impact of CD44 on coagulant activity was confirmed by using a visual coagulation assay previously optimized in the laboratory [23,24] (Figure 3B).

Previous works, including ours, have demonstrated the role of TF in providing tumor cells with enhanced abilities to accomplish early steps of metastasis (survival in the bloodstream and metastatic seeding). We thus examined the impact of CD44 silencing in a short-term experimental metastasis assay previously optimized in the laboratory to quantify early metastatic seeding in the lungs [23]. Comparing MDA-MB-231, Hs578T and EGF-treated MDA-MB-468 cells silenced for CD44 in vitro, we observed a clear diminution of human tumor cell content in lungs 24 h after injection (Figure 3C).

### 3.3. CD44 Regulates TF at a Transcriptional Level

**Figure 4:**
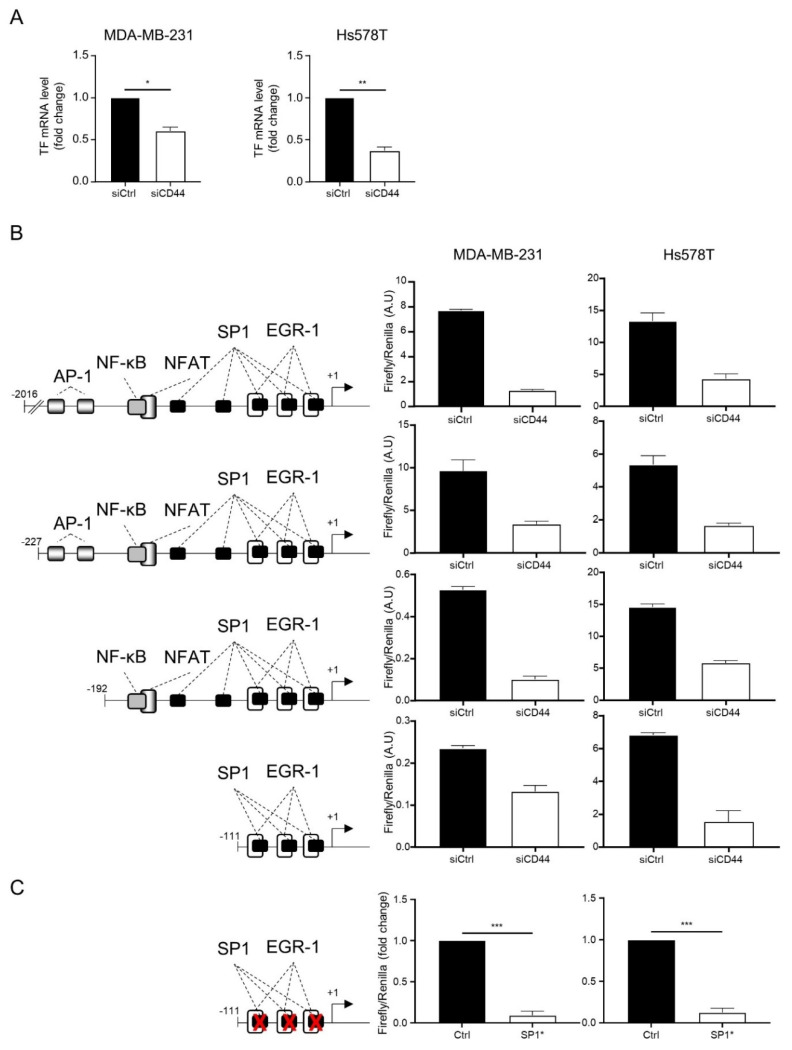
CD44 regulates TF expression at a transcriptional level: (**A**) RT-qPCR analyses of TF in EMT^+^ (MDA-MB-231 and Hs578T) cellular models transfected with a pool of 3 siRNAs directed against CD44 (siCD44) or 3 non-targeting siRNAs (siCtrl). (**B**) Luciferase reporter assays performed in MDA-MB-231 and Hs578T using TF promoter constructs harboring different size fragments of the TF promoter region (as illustrated in the left panel). A representative experiment is shown with results expressed in arbitrary units. (**C**) Luciferase reporter assays performed with a pGL-3-based luciferase reporter vector containing the -111 region of the TF promoter in its wild type form (Ctrl) or harboring a mutation in the Specificity Protein 1 (Sp1)/Early Growth Response factor 1 (EGR-1) overlapping binding sites (SP1*). Results normalized from 3 independent experiments are shown. *, *p* < 0.05; **, *p* < 0.01; ***, *p* < 0.001.

Considering the regulation of TF by CD44 evidenced here, we explored potential molecular mechanisms underlying this regulation. Altho ugh this does not exclude a potential post-translational regulation, we observed that CD44 silencing decreased TF mRNA levels in the two EMT^+^ cellular models examined (Figure 4A), rather directing our search towards a transcriptional regulatory mechanism. Literature data have shown that TF can be regulated in different cellular contexts by several transcription factors (including p65, Activator Protein 1 (AP1), Early Growth Response factor 1 (EGR-1) or Sp1) [15,39] which, as reported in independent literature, can mediate CD44 downstream signaling [42,43,44]. In order to pinpoint key transcription factors bridging CD44 to TF regulation, we used several TF promoter luciferase reporter vectors (generated in Pr. Mackman’s laboratory [39] and purchased at Addgene) harboring different length fragments of the TF promoter region (from -2016 pb to -111 pb, as cartooned in Figure 4B). We observed that CD44 silencing diminishes the luciferase activity of all vectors in MDA-MB-231 and Hs578T. These observations suggest that the smallest -111 pb TF promoter fragment contains regulatory elements that are sufficient to transmit the effect of CD44 silencing on TF regulation, and point to Sp1 and EGR-1 (which have three overlapping binding sites in this region) as potentially implicated transcription factors. Supportively, a -111 pb TF promoter reporter vector harboring mutations in the three Sp1 overlapping binding sites clearly had a lower activity than the control vector in both cell lines (Figure 4C).

**Figure 5:**
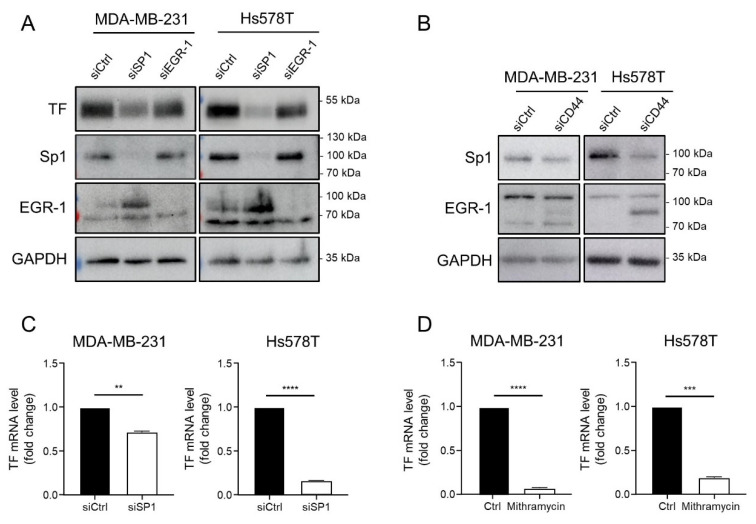
TF is regulated by CD44 at the transcriptional level through an Sp1 dependent mechanism: (**A**) Western blotting analyses of TF, Sp1, EGR-1 and GAPDH in EMT^+^ (MDA-MB-231 and Hs578T) cell lines transfected with a pool of 3 siRNAs directed against Sp1 (siSP1), EGR-1 (siEGR-1) or 3 non-targeting siRNAs (siCtrl) (original western blot images are shown in Supplementary Materials). (**B**) Western blotting analyses of Sp1, EGR-1 and GAPDH in EMT^+^ (MDA-MB-231 and Hs578T) cell lines transfected with a pool of 3 siRNAs directed against CD44 (siCD44) or 3 non-targeting siRNAs (siCtrl) (original western blot images are shown in Supplementary Materials). (**C**) RT-qPCR analyses of TF in EMT^+^ (MDA-MB-231 and Hs578T) cell lines transfected with a pool of 3 siRNAs directed against Sp1 (siSP1) or 3 non-targeting siRNAs (siCtrl). (**D**) RT-qPCR analyses of TF in EMT^+^ (MDA-MB-231 and Hs578T) cell lines treated or not (Ctrl) with mithramycin. **, *p* < 0.01; ***, *p* < 0.001; ****, *p* < 0.0001. (Figure S3: Uncropped Western blot figures).

Comparing the effect of Sp1 and EGR-1 silencing, we observed that siSP1 transfection most strongly diminished TF protein levels in the two cell lines (Figure 5A). Further emphasizing the importance of Sp1 in the CD44-TF regulatory axis, silencing CD44 diminished Sp1 levels, whereas EGR-1 protein levels rather increased (Figure 5B). Although observed in both cell lines, this effect was most obvious in Hs578T. Supportively, we also observed a diminution of TF mRNA levels after Sp1 silencing (Figure 5C) and after a treatment with mithramycin (Figure 5D) which, although not strictly specific, has been frequently used in the literature as a potent inhibitor of Sp1 activity [45,46].

These data taken together thus point to Sp1 as a key transcription factor implicated in a CD44/TF transcriptional regulatory axis.

## 4. Discussion

In our study, we identified a mechanism of TF regulation by CD44 in breast tumor cells which modulates their procoagulant activity.

Our results indeed show that: (i) CD44 and TF are co-expressed in different human breast EMT^+^ tumor cell models, (ii) silencing CD44 diminishes TF at the RNA and protein levels, (iii) silencing CD44 decreases the coagulant activity and the metastatic seeding properties of EMT^+^/TF^+^ tumor cells. Mechanistically, we show, using promoter reporter assays, that a short TF promoter sequence harboring Sp1 binding sites is sufficient to mediate a response to CD44 silencing, and that mutations of the Sp1 binding sites abolish the activity of this promoter sequence. Silencing Sp1 was supportively found to diminish TF levels.

Comparing different breast tumor cell systems of EMT, we thus first report here an association between CD44 and TF expression in EMT^+^ cellular backgrounds. The association of TF with EMT markers (enhanced vimentin expression, diminution of E-cadherin) was previously reported by Pr. Rak’s group [47] and by us in cell line models [23,24]. Our previous work also reported TF expression associated to vimentin expression in Triple Negative Breast Carcinoma (TNBC) but also in CTCs isolated from metastatic breast cancer patients [23]. On the other hand, the association between TF and CD44 reported here in EMT phenotypes is also in line with a massive body of literature that has been reviewed [5,26,27,28], functionally associating CD44 expression to EMT in a variety of tumor cell lines, in different cancer types and in human CTCs. In accordance with the recognized role of CD44 as a stem cell marker [29,30], few studies particularly established a relationship between TF expression and CSC phenotypes/signatures (CD133^+^, CD44^+^) in different cell lines, and the functional role of TF in providing tumor cells with stemness potential has also been reported [48,49,50,51,52]. TF has accordingly been proposed as a good target for strategies to eradicate tumor cells, and particularly CSCs. TF-targeting agents are thus being developed (e.g., drug-conjugated TF antibodies, CAR-NK) and some of which are currently being evaluated in clinical trials [48,49,53,54,55,56,57]. All-in-all, these data thus support that the overexpression of TF in tumor cells is associated with EMT and Cancer Stem Cell phenotypes, accordingly known to display important overlapping traits [31,58,59].

Complicating the scenario, CD44 is subjected to multiple splicing events and the relative importance of specific CD44 splice variants for particular tumor phenotypes has seemingly remained controversial [25,26,27,28], revealing important discrepancies related to tumor types and stages. Our sequencing analyses and western blotting patterns suggest that CD44s is the main variant associated with TF expression in the EMT^+^ breast tumor cells. CD44s was indeed the main variant detected in EMT^+^ MDA-MB-231 cells and Hs578T, as reported by others [60,61]. Sequence analyses of EMT^+^ Hs578T cells also identified CD44s as the major isoform, while western blot CD44 patterns clearly identified higher molecular weight CD44 forms in this cell line that could correspond to post-translational modifications. Interestingly, in the EMT iSnail-inducible system, a shift towards the CD44s isoform was particularly obvious. Such a shift towards CD44s induced by EMT transcription factors Snail or ZEB1 in breast tumor cell backgrounds has previously been reported by others [62,63]. In agreement with our observations, and as recently reviewed [27], literature data rather points to CD44s as a major pro-metastatic isoform in breast cancer contexts. For instance, Zhang and colleagues, performing a detailed genome-wide gene set enrichment analysis, confirmed by CD44 variants characterization in a TNBC cohort, demonstrated that CD44s associated with signatures of CSC and EMT whereas CD44v negatively correlated with these signatures.

Considering these observations together with our results, and although it is out of the scope of this manuscript to establish which specific CD44 isoform regulates TF expression, it feels safe to conclude that CD44s is the main isoform associated to a positive expression of TF in our different EMT^+^ breast tumor cell models.

More than a co-expression of TF and CD44 in EMT^+^ breast tumor cells, we identify here a regulatory link between the two molecules. Although this is still an underexplored area, different molecular mechanisms have been involved in TF upregulation in tumor cells, including various extracellular signals and signaling pathways, transcription factors or miRs [15,64]. More directly in line with our observations linking EMT and TF expression, Pr. Rak’s laboratory reported a modulation of TF expression induced along with an EMT phenotype through EGFR activation or E-cadherin blockade [65]. Our previous work also demonstrated that well-known EMT transcription factors, ZEB1 or Snail, contribute to regulate TF expression in the various EMT human breast tumor cells used in the present study [23]. We also reported that the intermediate filament vimentin is able to prevent a miR-dependent negative regulation of TF in breast tumor cell lines [24].

Mechanistically, our present data support a regulation of TF by CD44 at a transcriptional level. This is very well in line with the known role of CD44 as a signaling platform modulating various signaling pathways that modify the transcription of protumoral and prometastatic genes [27,32]. Silencing CD44 indeed decreased TF mRNA levels and the activity of a TF promoter luciferase reporter construct harboring a -2016 pb TF promoter sequence. This promoter fragment has been previously well characterized and shown to contain binding sites for various transcription factors including AP1, EGR-1, Sp1 or NF-κB [15], all of which have been shown to be potentially modulated by CD44 in independent literature data [42,43,44]. Several studies accordingly demonstrated the role of these transcription factors in regulating TF in various normal cell contexts (monocytes, endothelial cells) and also in tumor cells, particularly glioma or breast tumor cell lines [15,66,67]. The combined action of these different transcription factors thus seemingly fine tunes TF expression although specific transcription factors may take a lead in response to specific stimuli. Our results comparing the activity of different reporter vectors harboring truncated TF promoter sequences or mutated in the binding sites of well-known transcription factors point to Sp1 as an important transcription factor mediating the regulation of TF by CD44. The shorter -111 pb promoter sequence, which contains three Sp1 binding sites, was indeed still responsive to CD44 silencing, and mutating the three Sp1 sites diminished the construct activity. Supportively also, silencing CD44 was found to inhibit Sp1 and silencing Sp1 strongly diminished TF mRNA levels. It is worth mentioning that silencing EGR-1 (for which three binding sites overlapping with the three Sp1 binding sites are found in the -111 pb TF promoter construct) also inhibited TF expression but to a much lesser extent than Sp1 silencing. In support of our results, Nam and colleagues similarly reported a strong decreased expression of Sp1 after CD44 silencing in MDA-MB-231 and Hs578T breast tumor cells, that was associated with decreased migratory/invasive properties [42]. It is important to emphasize that the diminution of TF after CD44 silencing or Sp1 silencing is less pronounced in MDA-MB-231 cells than in Hs578T cells. It may well be that the CD44-Sp1 axis in this cell line is not as potent to regulate TF expression as compared to what occurs in Hs578T cells. It is also worth noting that the basal level of TF is much higher in MDA-MB-231 cells than in the Hs578T cells, suggesting a regulation by multiple effectors. This may also partly explain that affecting a single effector pathway may have only limited impact on TF levels.

In addition, our results also support a functional role of this CD44/TF regulatory axis in providing tumor cells with enhanced procoagulant activity and increased metastatic colonization abilities. Silencing CD44 indeed inhibited the coagulant properties of tumor cells which could contribute to the decreased ability of tumor cells to accomplish metastatic colonization when injected as CTCs in experimental metastasis assays. Supportively, independent sets of data including ours, show, on one hand, that EMT-shifted CTCs represent a subpopulation of CTCs with enhanced metastatic competence [5,11,12,13] and, on the other hand, that TF-dependent coagulant properties of tumor cells facilitate their survival in the blood stream and metastatic colonization [15,21,22,68].

Overall, our results here have identified a regulatory pathway bridging CD44 to TF expression in EMT^+^/TF^+^ breast tumor cells which functionally modulate their coagulant properties. Although the data presented here remain at a basic research level, deciphering regulatory pathways and identifying molecular intermediates bridging different molecular actors may open up new therapeutic opportunities and options. In addition, this may also contribute to better delineate tumor subphenotypes and define clinically more robust predictive or prognostic companion biomarkers. In addition, the analysis of CD44 and TF in clinical studies may help specify their roles in the occurrence of cancer-associated thromboembolism, which remains a leading cause of cancer-related death [14,69,70,71].

## 5. Conclusions

TF expression thus appears finely regulated in tumor cells and several EMT pathways may thus converge to induce its expression. Our present findings identifying a regulation of TF by CD44 further extend these observations to the context of CSC phenotypes. Such a mechanism of regulation of TF by CD44 could thus contribute to provide EMT^+^/CSC phenotypes with higher coagulant properties, which are known to facilitate early colonization processes.

## Data Availability

The data presented in this study are available on request from the corresponding author.

## Supplementary Materials

The following supporting information can be downloaded at: https://www.mdpi.com/article/10.3390/cancers14133288/s1, Figure S1: TF and CD44 coexpress in EMT cellular contexts. FACS analyses and associated Mean Fluorescence Intensity (MFI) quantifications of CD44 and TF in EMT^+^ (MDA-MB-231 and Hs578T) vs EMT^−^ (MCF7) and in EMT-inducible (MDA-MB-468 treated or not (Ctrl) with EGF to induced EMT) cell lines, Figure S2: Validation of the enzymatic fluorescent coagulation assay Enzymatic fluorescent coagulation assays performed in EMT^+^ (MDA-MB-231 and Hs578T) vs EMT^−^ (MCF7 and T47D), and in EMT^+^ (MDA-MB-231) cell lines transduced with a shRNA directed against TF (shTF) or a non-targeting shRNA (shCtrl). Fluorescence intensity was followed during 8 h, Figure S3: Original uncropped images of western blots and densitometric analyses. Table S1: Overview of Study Design, Table S2: list of siRNA [72,73,74], Table S3: list of primer sequences used for qPCR analyses, Table S4: list of antibodies used for western blotting analyses, Table S5: list of antibodies used for FACS analyses, Table S6: list of primer sequences used for sequencing, Table S7: list of plasmids used for dual-luciferase reporter assays [39].
