# Supplementary material for: Regulation of Tissue Factor by CD44 Supports Coagulant Activity in Breast Tumor Cells
Source: Cancers (Basel). 2022 Jul 5;14(13):3288. doi: 10.3390/cancers14133288 (PMC9266039; doi:10.3390/cancers14133288)
Supplement: Supplementary file 1 [file cancers-14-03288-s001.zip › cancers-1796468-supplementary.pdf]

cancers MDPI

Suplementary Material

Regulation of Tissue Factor by CD44 Supports Coagulant Activity in Breast Tumor Cells

Amélie V. Villard [1,†], Anthony Genna [1,†], Justine Lambert [1], Marianna Volpert [2], Agnès Noël [1], Brett Hollier [2], Myriam Polette [3], Aline M. Vanwynsberghe [1] and Christine Gilles [1,*]

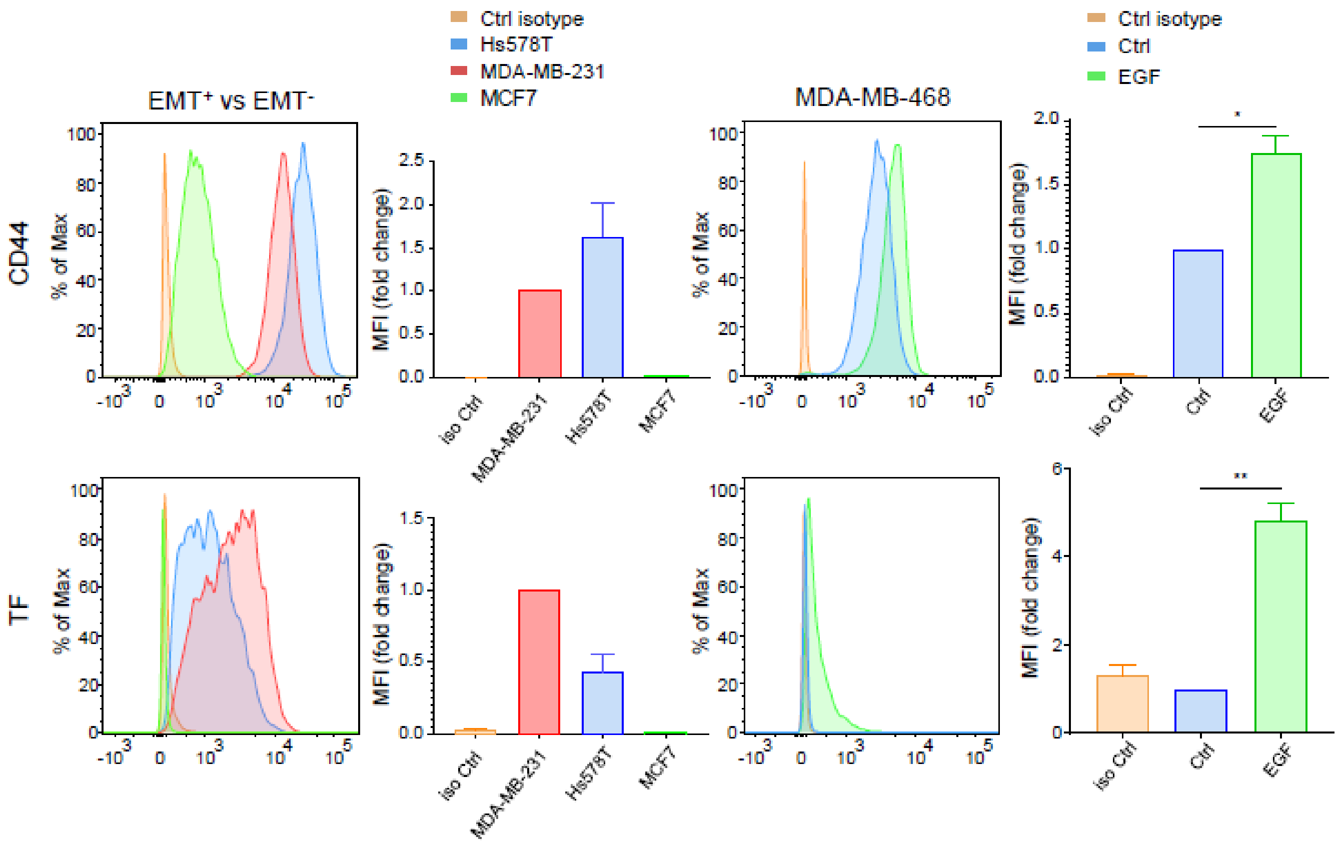

Figure S1. TF and CD44 coexpress in EMT cellular contexts. FACS analyses and associated Mean Fluorescence Intensity (MFI) quantifications of CD44 and TF in EMT+ (MDA−MB−231 and Hs578T) vs EMT- (MCF7) and in EMT−inducible (MDA−MB−468 treated or not (Ctrl) with EGF to induced EMT) cell lines. *, $p < 0.05$; **, $p < 0.01$.

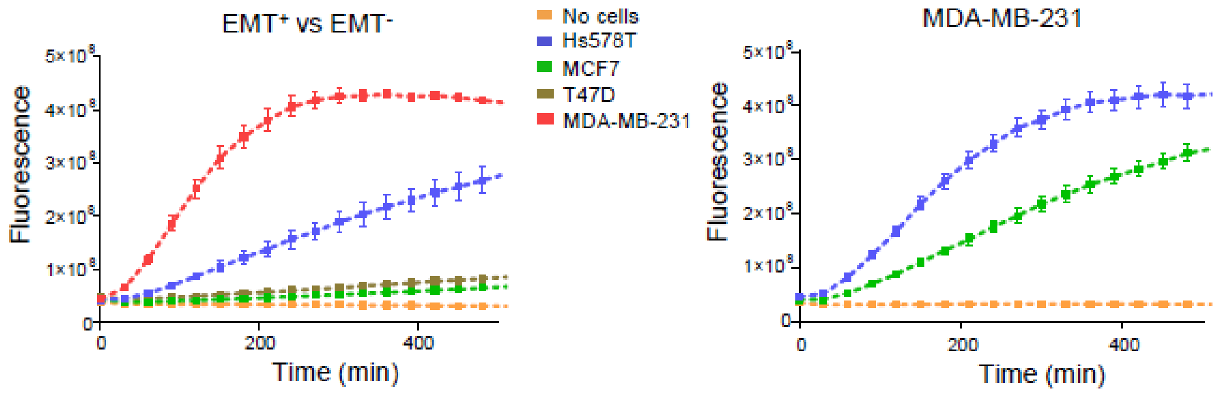

Figure S2. Validation of the enzymatic fluorescent coagulation assay. Enzymatic fluorescent coagulation assays performed in EMT⁺ (MDA–MB–231 and Hs578T) vs EMT⁻ (MCF7 and T47D), and in EMT⁺ (MDA–MB–231) cell lines transduced with a shRNA directed against TF (shTF) or a non–targeting shRNA (shCtrl). Fluorescence intensity was followed during 8 hrs.

Blots for Figure 1A

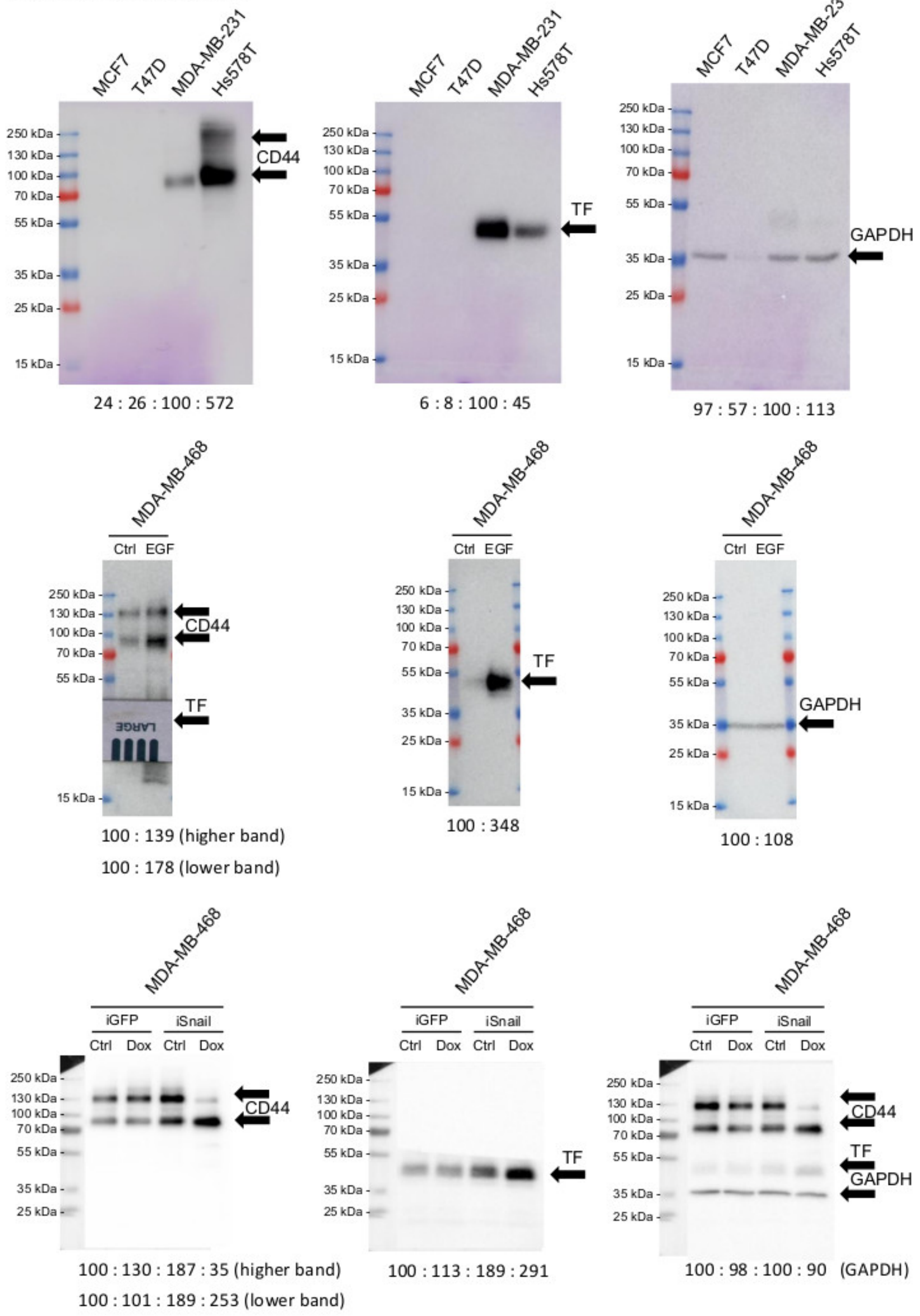

Blots for Figure 2A

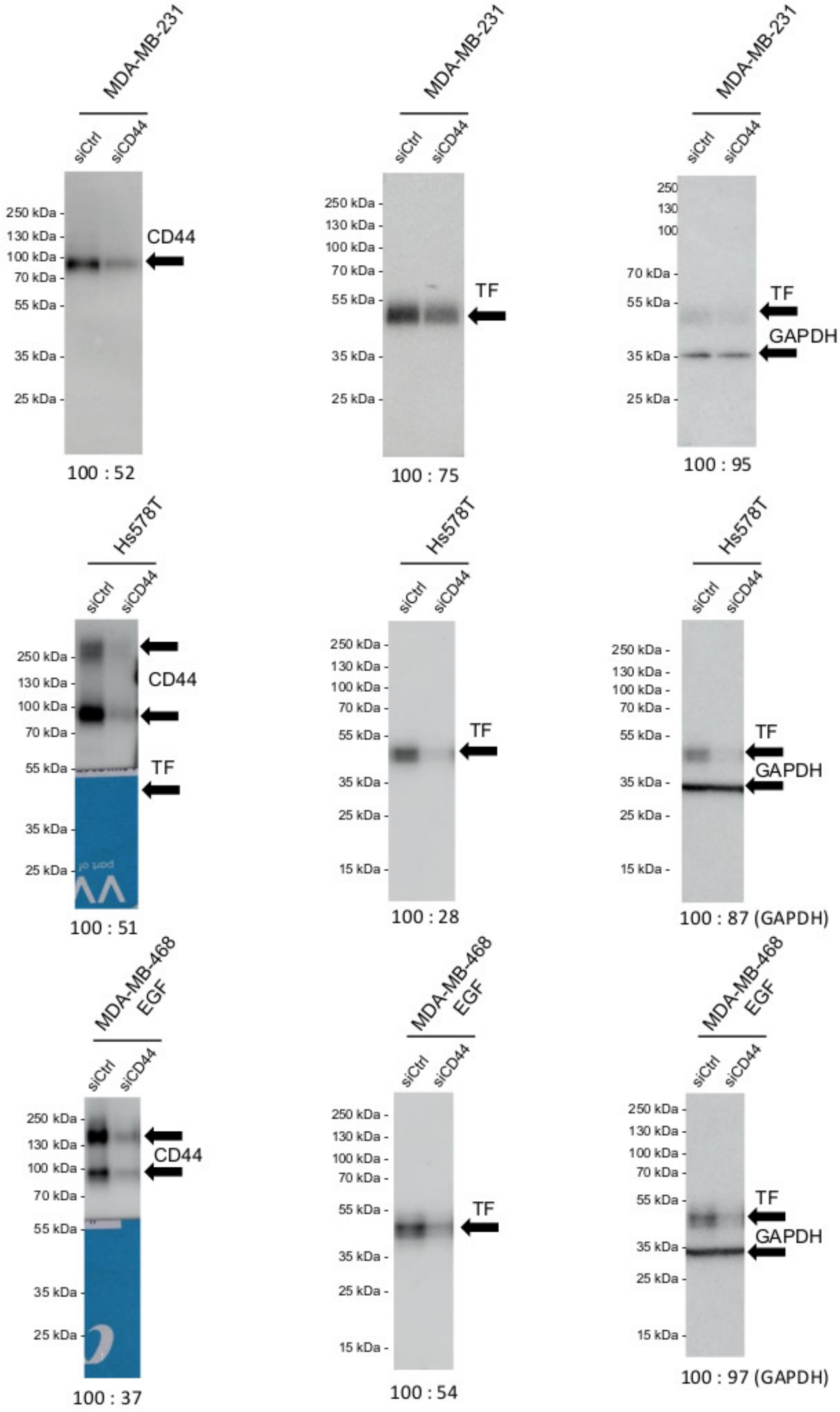

Blots for Figure 5A

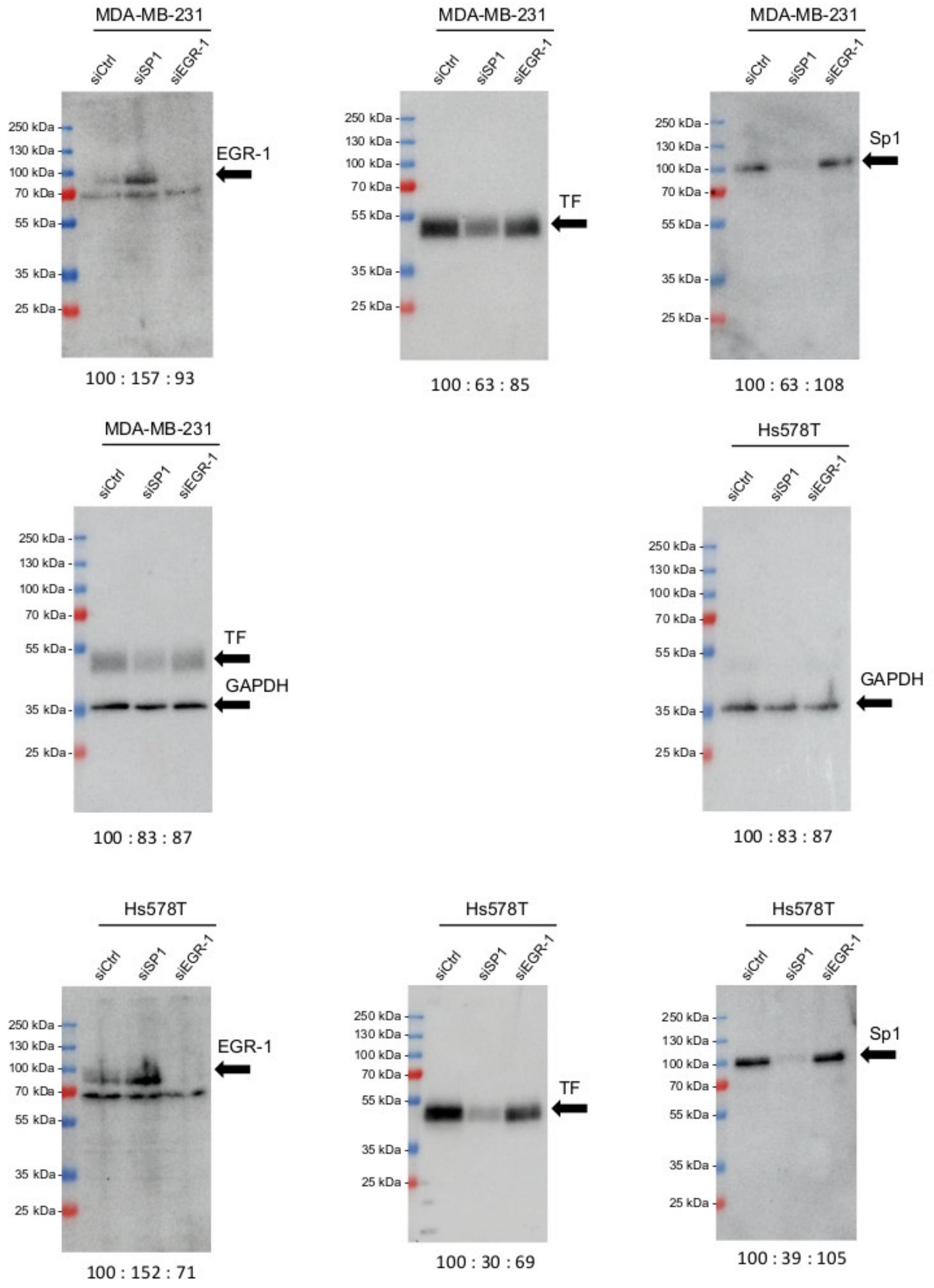

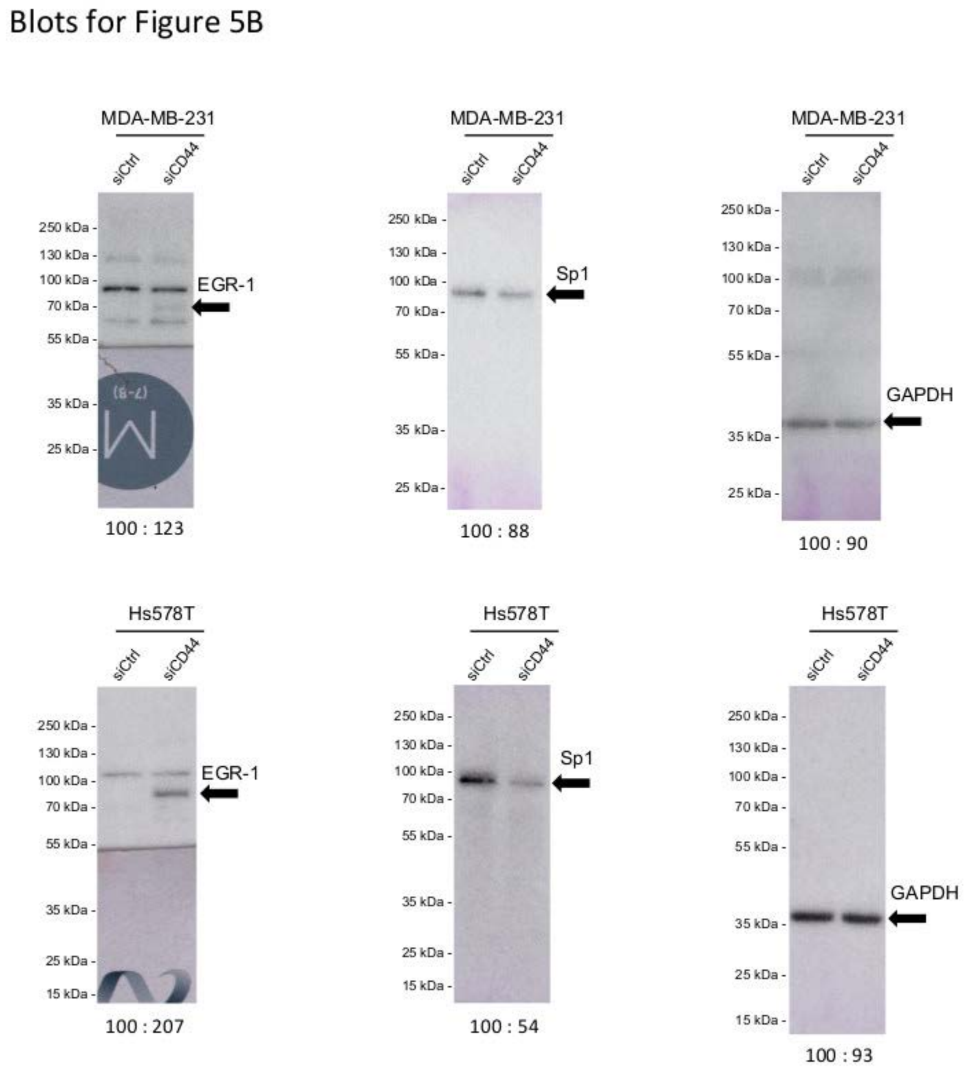

Figure S3. Uncropped Western blot figures

Table S1. Overview of Study Design.

Analyses	Methods
CD44 and TF expression in EMT+ breast tumor cell lines	FACS, Western blotting for TF and CD44 on MDA−MB−231, Hs578T, MDA−MB−468 (induced or not to EMT).
Impact of CD44 silencing on TF expression	FACS, Western blotting for TF and CD44 after CD44 siRNA transfection in MDA−MB−231, Hs578T, MDA−MB−468 (induced or not to EMT).
Impact of CD44 silencing on coagulant properties	Enzymatic fluorescent coagulation assays and visual clot assays on MDA−MB−231, Hs578T, MDA−MB−468 (induced or not to EMT) transfected with CD44 siRNA.
Impact of CD44 silencing on metastatic seeding	Experimental metastasis assays on MDA−MB−231, Hs578T, MDA−MB−468 (induced or not to EMT) transfected with CD44 siRNA
Analysis of a TF transcriptional regulation by CD44	TF−promoter reporter assays<br>Western blotting and RT−qPCR (for TF or Sp1 or EGR−1) after CD44 silencing, Sp1 silencing or mithramycin treatments in MDA−MB−231 cells and Hs578T cells.

Table S2. list of siRNA.

siRNA	Sequences
siCtrl 1	5'−CAGAACUAGAUUGCGAAUA−3'
siCtrl 2	5'−GCCCAAAUAAUGGUCAGAA−3'
siCtrl 3	5'−GAUACUAUCUAGUCUAGAC−3'

siCD44 1 [1]	5'−GCAGAUCGAUUUGAAUAUA−3'
siCD44 2 [1]	5'−GUAUGACACAUAUUGCUUC−3'
siCD44 3 [2]	5'−AAAUGGUCGCUACAGCAUC−3'
siSP1 1 [3]	5'− GCAACAUGGGAAUUAUGAA−3'
siSP1 2 [3]	5'−GGCAGACCUUUACAACUCA −3'
siSP1 3 [3]	5'−CCACAAGCCCAAACAAUCA−3'
siEGR1 1*	5'− CGACAGCAGUCCCAUUUAC −3'
siEGR1 2*	5'− GGACAUGACAGCAACCUUU −3'
siEGR1 3*	5'− GACCUGAAGGCCCUCAAUA −3'

* from Dharmacon™.

Table S3. list of primer sequences used for qPCR analyses.

Genes	Primers	Sequences
hFT	Forward	5'−CAGACAGCCCGGTAGAGTGT−3'
	Reverse	5'−CCACAGCTCCAATGATGTAGAA−3'
hGAPDH	Forward	5'−TGCCGTCTAGAAAAACCTGCCAAA−3'
	Reverse	5'−CTCTCTTCCTCTTGTGCTCTTGCT−3'
hGAPDH (nested−qPCR)	Forward	5'−ACCCTACTGATGATGACGTGAGCA−3'
	Reverse	5'−TGGAATGTGTCTTGGTCTCTGGTA−3'
mGAPDH	Forward	5'−TGTCCGTCGTGGATCTGAC−3'
	Reverse	5'−GAGTTGCTGTTGAAGTCGCA−3'

Table S4. list of antibodies used for western blotting analyses.

Anticorps	Clone	Source	References
Mouse anti−human tissue factor	VD8	Sekisui Diagnostics	ADG4508
Mouse anti−human CD44	2C5	R&D Systems	BBA10
Rabbit anti−human Sp1	Polyclonal	Cell Signaling	5931
Rabbit anti−human EGR−1	15F7	Cell Signaling	4153
Mouse anti−human GAPDH	6C5	Merck Millipore	MAB374
Goat anti−rabbit/HRP	Polyclonal	Cell Signaling	7074
Horse anti−mouse/HRP	Polyclonal	Cell Signalling	7076

Table S5. list of antibodies used for FACS analyses.

Anticorps	Clone	Source	References
Alexa fluor® 488 anti−mouse/human CD44	Monoclonal Rat IgG2b, κ Clone IM7	Biolegend	103015
Alexa fluor® 594 anti−human TF	Monoclonal Mouse IgG1 Clone −#323519	R&D Systems	FAB23391T
Alexa Fluor® 488 Isotype Ctrl Anti-body	Monoclonal Rat IgG2b, κ	Biolegend	400625
Alexa Fluor® 594 Isotype Ctrl Anti-body	Monoclonal Mouse IgG1	R&D Systems	FAB110T

Table S6. list of primer sequences used for sequencing.

Genes	Primers	Sequences
hCD44	Forward	5'−_GCAGCACTTCAGGAGGTTACATCT−3'
	Reverse	5'− TGTGGACATGAAGATTGGGGTGTAA−3'

Table S7. list of plasmids used for dual−luciferase reporter assays.

Plasmids	Regions	Source	References
− 2106	−2106 nt to +121 nt	Addgene	15440 [4]
−227	−227 nt to +121 nt	Addgene	15442 [4]
−192	−192 nt to +121 nt	Addgene	15446 [4]
−111	−111 nt to +121 nt	Addgene	15447 [4]

Reference

1. Li, C.Z., Liu, B., Wen, Z-Q., Li, HY., Inhibition of CD44 expression by small interfering RNA to suppress the growth and metastasis of ovarian cancer cells in vitro and in vivo. Folia Biol (Praha), 2008. **54**(6): p. 180-6.
2. Singleton, P.A., Salgia, R., Moreno-Vinasco, L., Moitra, J., Sammani, S., Mirzapoiazova, T., Garcia, J.G.N., *CD44 regulates hepatocyte growth factor-mediated vascular integrity. Role of c-Met, Tiam1/Rac1, dynamin 2, and cortactin.* J Biol Chem, 2007. **282**(42): p. 30643-57.
3.] Bin, L., Kim, B.E., Hall, C.F., Leach, S.M., Leung, D.Y.M., Inhibition of transcription factor specificity protein 1 alters the gene expression profile of keratinocytes leading to upregulation of kallikrein-related peptidases and thymic stromal lymphopoietin. J Invest Dermatol, 2011. **131**(11): p. 2213-22.
4. Mackman, N., *Regulation of the tissue factor gene.* FASEB journal : official publication of the Federation of American Societies for Experimental Biology, 1995. **9**(10): p. 883-9.
